# Genetic structure and gene flow of the flea *Xenopsylla cheopis* in Madagascar and Mayotte

**DOI:** 10.1186/s13071-017-2290-6

**Published:** 2017-07-20

**Authors:** Mireille Harimalala, Sandra Telfer, Hélène Delatte, Phillip C. Watts, Adélaïde Miarinjara, Tojo Rindra Ramihangihajason, Soanandrasana Rahelinirina, Minoarisoa Rajerison, Sébastien Boyer

**Affiliations:** 1 Medical Entomology Unit, Institut Pasteur of Madagascar, Ambatofotsikely, PO box 1274, 101 Antananarivo, Madagascar; 2 School of Biological Sciences, University of Aberdeen, Aberdeen, AB24 2TZ UK; 3 UMR PVBMT, CIRAD, 7 Chemin de l’IRAT, Saint Pierre, La Réunion France; 4 Department of Ecology and Genetics, University of Oulu, FI-90014 Oulu, Finland; 5 Plague Unit, Institut Pasteur of Madagascar, Ambatofotsikely, PO box 1274, 101 Antananarivo, Madagascar

**Keywords:** Madagascar, Mayotte, *Xenopsylla cheopis*, Microsatellites, Genetic structure, Gene flow, Plague

## Abstract

**Background:**

The flea *Xenopsylla cheopis* (Siphonaptera: Pulicidae) is a vector of plague. Despite this insect’s medical importance, especially in Madagascar where plague is endemic, little is known about the organization of its natural populations. We undertook population genetic analyses (i) to determine the spatial genetic structure of *X. cheopis* in Madagascar and (ii) to determine the potential risk of plague introduction in the neighboring island of Mayotte.

**Results:**

We genotyped 205 fleas from 12 sites using nine microsatellite markers. Madagascan populations of *X. cheopis* differed, with the mean number of alleles per locus per population ranging from 1.78 to 4.44 and with moderate to high levels of genetic differentiation between populations. Three distinct genetic clusters were identified, with different geographical distributions but with some apparent gene flow between both islands and within Malagasy regions. The approximate Bayesian computation (ABC) used to test the predominant direction of flea dispersal implied a recent population introduction from Mayotte to Madagascar, which was estimated to have occurred between 1993 and 2012. The impact of this flea introduction in terms of plague transmission in Madagascar is unclear, but the low level of flea exchange between the two islands seems to keep Mayotte free of plague for now.

**Conclusion:**

This study highlights the occurrence of genetic structure among populations of the flea vector of plague, *X. cheopis*, in Madagascar and suggests that a flea population from Mayotte has been introduced to Madagascar recently. As plague has not been reported in Mayotte, this introduction is unlikely to present a major concern for plague transmission. Nonetheless, evidence of connectivity among flea populations in the two islands indicates a possibility for dispersal by fleas in the opposite direction and thus a risk of plague introduction to Mayotte.

**Electronic supplementary material:**

The online version of this article (doi:10.1186/s13071-017-2290-6) contains supplementary material, which is available to authorized users.

## Background

The oriental rat flea, *Xenopsylla cheopis* (Siphonaptera: Pulicidae) is a holometabolous insect ectoparasite and was first described in Egypt [1], which is believed to represent its origin [2]. This species of flea is now cosmopolitan because of widespread dispersal (principally on ships) by its preferred rodent host, the black rat *Rattus rattus* (Rodentia: Muridae) [3]. *Xenopsylla cheopis* is also a frequent parasite on the brown rat *R. norvegicus* and can parasitize other small mammals [4, 5]. This flea draws particular attention because of its role as a vector of pathogens responsible for human diseases such as plague and murine typhus [6, 7]. Indeed, it is thought to be the most efficient vector of the plague bacterium, *Yersinia pestis* [8] and can transmit the plague both between rodent hosts and to humans.

Plague is a re-emerging disease occurring in many regions of the World [9] but, more than 90% of worldwide cases in 2014–2015 were reported in Africa [9]; Madagascar being the most affected country [10]. Plague was introduced to the eastern coastal region of Madagascar in 1898 [11], apparently by ships from India [12] and subsequently, spread to other ports before reaching the Central Highlands in 1921, where it became endemic [13] inside the “plague focus” (altitude >800 m) [14]. Nevertheless, some areas outside this focus have epidemics, such as the District of Ikongo (altitude ~750 m) in 1998 [15, 16] and the District of Ambilobe (altitude <500 m) in 2011 [17]. Although apparently absent from coastal areas since the 1930s, plague re-emerged in Mahajanga, a port in the north-west in 1991 with annual outbreaks between 1995 and 1999 [13]. Eighty-six per cent of suspected cases reported in Madagascar between 2007 and 2011 were classified as bubonic plague [14], reflecting the important role played by flea vectors in the transmission cycle.

In Madagascar, *Rattus rattus*, the principal plague reservoir is abundant and found in diverse habitats [18]. Two flea species (Pulicidae) are reported to be the main vectors of *Y. pestis*: *Xenopsylla cheopis* [19], and an endemic species *Synopsyllus fonquerniei* [20]. *Xenopsylla cheopis* can be found throughout most of Madagascar, independent of altitude [21], while *S. fonquerniei* is largely absent below 800 m [22]. Unlike *S. fonquerniei*, *X. cheopis* is predominantly found on *R. rattus* captured inside houses [15, 18, 22] and is therefore thought to be an important vector for human cases. Population genetic and phylogeographic studies have provided important insights into the invasion history and population ecology of *R. rattus* and *Y. pestis* in Madagascar. A large nationwide study of *R. rattus* indicated the existence of only two clearly defined genetic groups corresponding to two separate introduction events, with one largely confined to the far north of the island and one reflecting a large spatial expansion of an introduction that occurred in the south [23]. In contrast, *Y. pestis* exhibited significant geographical separation among 15 identified subclades, implying largely local epidemiological cycles with limited gene flow [24]. However, the same study also found evidence of long distance transfers, probably human-mediated [24].

Despite the importance of *X. cheopis* for plague transmission in Madagascar, no studies of its population genetic structure have been conducted. Most studies of *X. cheopis* involve laboratory studies of the flea-bacteria relationship [8], the host-flea relationship [25] or insecticide treatment effects [26–28]. Field studies on population structure and dispersal in Madagascar and surrounding areas are required to improve our understanding of vector dynamics and the associated epidemiological risks.

For example, studies of natural populations of the plague flea vector *Oropsylla hirsuta*, which transmits plague bacterium to the black-tailed prairie dog, highlighted the lack of isolation by distance and spatial genetic structure of the flea, and demonstrated that re-colonization of fleas from plague-free to plague zones occurred and caused a flea population expansion after epizootics [29]. The implication is that this flea has reasonable dispersal ability at this spatial scale, with the high estimated rate of gene flow exhibited by *O. hirsuta* potentially associated with dispersal by its hosts (prairie dogs and other mammals) [30].

Although the population genetic structure of parasites is often linked to the population genetic structure and dispersal of their hosts, the extent of this congruence will depend on the intimacy of the parasite-host association [31], and factors such as host specificity and time spent on host. In many cases, parasites are expected to show stronger structuring than their hosts due to limited dispersal abilities and lower effective population sizes [32]. A comparative study of the fur flea *Listropsylla agrippinae* and the nest flea *Chiastopsylla rossi* revealed different phylogeographic patterns, with the nest flea showing higher genetic divergence between sampling localities, presumably due to more restricted dispersal as a consequence of less time spent on hosts [33]. In other cases, a lack of concordance in the phylogeographic and population genetic structure between some flea species and their hosts suggests the potentially important role of dispersal by other sympatric hosts [30, 34]. As a fur flea, adult *X. cheopis* are predicted to have relatively frequent opportunities to be dispersed by their hosts. However, in Madagascar, unlike its principal host, *X. cheopis* is strongly geographically restricted to houses [22] and is therefore spatially restricted to a small subset of *R. rattus* populations. Other mammals that are less abundant than *Rattus rattus*, such as *Mus musculus* and *Suncus murinus*, can be infested with *X. cheopis* [22]. However, unlike *R. rattus*, these are peridomestic and largely restricted to around houses. Thus, we predict that *X. cheopis* populations will show much greater genetic structure than *R. rattus* populations due to a combination of limited dispersal and lower effective population sizes.

The dispersal of rodents and their fleas from the plague focus in Madagascar poses a serious potential health threat to other areas of Madagascar and to neighboring islands, such as Mayotte. Plague has not been reported in Mayotte, however, there is an important maritime trade route between Mayotte and north-west Madagascar. Our objectives were therefore: (i) to determine the genetic diversity and spatial genetic structure of *X. cheopis* populations in Madagascar; (ii) to determine the extent and pattern of any gene flow between *X. cheopis* populations in Madagascar and Mayotte, which may constitute an indication of plague introduction risk.

## Methods

### Specimen sampling

**Fig. 1:**
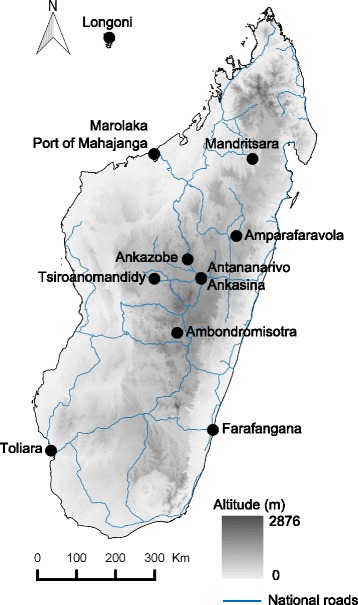
Sampling sites of the flea specimens used. Grey color variation of map corresponds to altitude variation. There were two sites in Mahajanga (Port and Marolaka) and two sites in Antananarivo (Antananarivo and Ankasina)

Samples of *X. cheopis* were collected in two areas of Mahajanga, Madagascar (the Port of Mahajanga and Marolaka) and the village of Longoni in Mayotte (Fig. 1). Additional specimens of *X. cheopis* were available from a further nine sites in Madagascar. All fleas were collected from small mammal hosts that had been trapped following published protocols [35]. Some specimens in Madagascar were collected as part of human plague outbreak responses and/or rodent-vector monitoring (Table 1). Fleas were collected alive on their hosts and preserved in 70% ethanol. Two hundred and five fleas were collected (Table 1) and species identification was performed by an expert taxonomist. When available, more than 10 specimens per site were analysed genetically.

**Table 1:** Sample sites, abundance of rats and fleas and variation in basic genetic diversity parameters

Sites	Longitude	Latitude	Year of sampling	Plague epidemic	No. of fleas (No. of rodents)	Flea index^a^	Na	He	Ho	F_IS_	*P*
Mayotte											
Longoni	45.159	-12.733	2014	No	39 (16)	0.5	2.56	0.32	0.25	0.24*	< 0.0001
Madagascar											
Marolaka	46.314	-15.725	2014	No	46 (13)	na	4.44	0.50	0.41	0.19*	< 0.0001
Port of Mahajanga	46.309	-15.725	2014	No	13 (3)	0.7	3.11	0.44	0.41	0.11	0.075
Ankasina	47.512	-18.910	2014	Yes	16 (6)	3.5	2.89	0.44	0.33	0.28*	< 0.0001
Amparafaravola	48.416	-17.833	2015	Yes	20 (4)	7.8	3.00	0.42	0.37	0.16*	0.008
Ankazobe	47.169	-18.434	na	Yes	21 (7)	0.7	3.11	0.44	0.30	0.34*	< 0.0001
Tsiroanomandidy	46.316	-18.933	2013	Yes	19 (na)	5.4	2.22	0.25	0.27	-0.06	0.770
Antananarivo	47.516	-18.933	1996	na	9 (na)	na	2.43	0.33	0.07	0.79*	< 0.0001
Toliara	43.666	-23.350	2012	No	5 (1)	10.3	1.78	0.26	0.29	0	0.550
Farafangana	47.816	-22.816	2012	No	5 (1)	1.4	1.78	0.28	0.36	-0.14	0.587
Mandritsara	48.830	-15,852	2014	Yes	7 (na)	0.3	2.78	0.43	0.24	0.51*	< 0.0001
Ambondromisotra	46.903	-20.323	2013	Yes	5 (na)	1.7	2.11	0.30	0.29	0.14	0.248

*Abbreviations*: *Na* average number of alleles per locus, *He* expected heterozygosity, *Ho* observed heterozygosity, *F*_*IS*_ inbreeding coefficient, *P* *P*-value for F_IS_, *na* not available

^a^The flea index is the average number of fleas per rat. A flea index >1 represents a potential plague risk

*Significant F_IS_ values; Hardy-Weinberg exact test: *P* < 0.05

### Isolation of microsatellite loci

Genomic DNA was extracted using a DNeasy Blood and Tissue Kit (Qiagen, Hilden, Germany) from 25 *X. cheopis* fleas collected in Madagascar. Partial genomic libraries enriched for microsatellite repeats were constructed using the protocol described by [36, 37] and summarized in Additional file 1. After an initial screening of 44 candidate microsatellite loci for suitability for genotyping (Additional file 1), a panel of 12 loci were deemed suitable for genotyping based on ease of scoring (e.g. no spurious PCR products, few stutter bands) and apparent polymorphism (Table 2).

**Table 2:** Summary of the microsatellite loci and PCR primers developed for the flea *Xenopsylla cheopis*

Multiplex pool	Primer (locus) names	Primer sequences (5′–3′)	Repeat motifs	Size range (bp)	Allele number
Pool 1	XC024	F: 6-FAM-ATGCAGCTCGTTCGTCTCC	CA(5)...CA(13)...CA(5)	ind	–
		R: GTCCAATTCATCCGCATCG			
	XC009	F: NED-CATTGCGGGAGCATCAG	(CA)5...(CA)8	283–303	6
		R: TGCAGGCACAAAATTCGAC			
	XC018	F: PET-TCGATTCAGCCGTTTTCG	(CA)10	ind	–
		R: TTGGAGAAGGAGATGTGTATGC			
	XC037	F: VIC-GGGCCACCGAGTTGACG	[(GTT)2...]13	290–348	4
		R: TGGTGTTCCGTTACCGTTCC			
Pool 2	XC007	F: 6-FAM-CTGGTTGGATTGTCTCC	(CA)5...(CA)22	ind	–
		R: ACTATGCCGGATTAAGG			
	XC021	F: PET-AGTGGACCGAGAACAGAGC	(GT)9	242–258	5
		R: TCATGTAAAGAGACCTGAGACC			
	SF009	F: NED-CGTGTAGTTGCGAGAGAAGC	(GT)4...(GT)5	168–187	5
		R: GGAGAAGTGCGTTTACAGAGC			
	XC013	F: VIC-CAAAATTGGAGAAGGAGACG	[GT(2)..GTAT]5	164–235	5
		R: AAATCGTTGACGGAAGAAGC			
Pool 3	XC023	F: PET-CTAGTAAACGCAAACGCTACC	[GT(3)...]7	300–380	5
		R: CCCCCAAACAAATCAGC			
	XC044	F: 6-FAM-AAAAGTAAAGTCGAACAAGTGG	(GTAT)6...(GT)5...(GTAT)8	396–428	5
		R: GCTTATAGGTTACAAACATCTGG			
	XC016	F: NED-ATCGACCCCAAAATCAGC	GT(10)...GTAT(12)...GT(7)	370–400	11
		R: ACCCCTGGTTGGATTGC			
	XC002	F: VIC-GCAGGCACAAAATTCGACA	GT(9)...GT(5)	266–302	5
		R: GCGGGGGCATCAGTTAAT			

*Abbreviation*: ind, pairs of primers tested did not provide specific pics in the targeted locus

### DNA extraction, PCR and genotyping

Genomic DNA for genotyping was extracted using the Instagene™ Matrix kit (Bio-Rad Laboratories Inc., California, USA) following the manufacturer’s instructions. Multiplex PCR were performed by pooling microsatellite primers into three pools of 4 pairs of primers per pool (Table 2). Multiplex PCRs contained 7.5 μl of Type-it microsatellite PCR kit (2×) (Qiagen, Hilden, Germany), 0.4 μl of each forward and reverse primers of the pool (10 μM), 1 μl of DNA sample and sufficient water for a 15 μl final reaction volume. Thermal amplification conditions were 95 °C for 5 min, followed by 35 cycles of 95 °C for 30 s, 58 °C for 45 s, 72 °C for 55 s and a final extension step of 72 °C for 10 min. PCR products were sized by capillary electrophoresis on an ABI prism 3130 (Applied Biosystems, California, USA) with GeneScan™ 500 LIZ® Size Standard (Applied Biosystems, California, USA) and GENEMAPPER software (Applied Biosystems, California, USA).

### Statistical analysis

Genotypic linkage disequilibrium between pairs of loci was tested with the exact test implemented in the GENEPOP v.4.3 [38]. The same software was used to estimate basic measures of genetic variability: mean number of alleles per locus (Na), observed (Ho) and expected (He) heterozygosities and F_IS_ [39] for each population. Significance test for F_IS_ values were performed using Hardy-Weinberg exact test implemented in GENEPOP. Allelic data were checked for null alleles, allelic dropout and stutter bands using MICROCHECKER v.2.2.3 with the Oosterhout algorithm [40]. Population differentiation (F_ST_) was estimated using ARLEQUIN v.3.5.2.1 [41]; the ENA method was used also to calculate an unbiased F_ST_ (F_ST_^ENA^) using FREENA [42].

### Identification of genetic clusters

We used two approaches to determine the number of distinct genetic populations: a Bayesian clustering approach using STRUCTURE [43] and the discriminant analysis of principal components (DAPC) [44].

STRUCTURE simultaneously identifies potential populations (clusters) and probabilistically assigns individuals to each of the *K* populations based on the sample genotypes. STRUCTURE runs were performed using the admixture model and correlated allele frequencies, with one million iterations of the Markov Monte Carlo Chain (MCMC) used as ‘burn-in’ that were followed by ten million MCMC iterations; the probability to observe the data [Ln P(D)] was calculated for values of K ranging from 1 to 12, with five iterations for each K-value. The best estimate of K was taken to be the maximum value observed before the plateau of the curve Ln P(D) against K [43]. STRUCTURE HARVESTER [45] was also used to identify the most pronounced level of population structure using the method of Evanno et al. [46]. CLUMPP v.1.1.2 [47] was used to find the optimal alignment from replicate STRUCTURE runs, with the summary of results generated using DISTRUCT v.1.1 [48].

DAPC is a multivariate statistical method which uses the *k*-means clustering approach [44]. DAPC first transforms data using a principal components analysis (PCA) and subsequently identifies clusters using discriminant analysis (DA). This method thus defines genetic clusters, assigns individuals to clusters and allows visual assessment between-population differentiation. DAPC was implemented using the *adegenet* package [49] of the R software [50]. The identification of the number of genetic clusters was done using the function *find.clusters* with the prior that the maximum number of clusters is equal to 12 (K = 12).

### Inference of population introduction

**Fig. 2:**
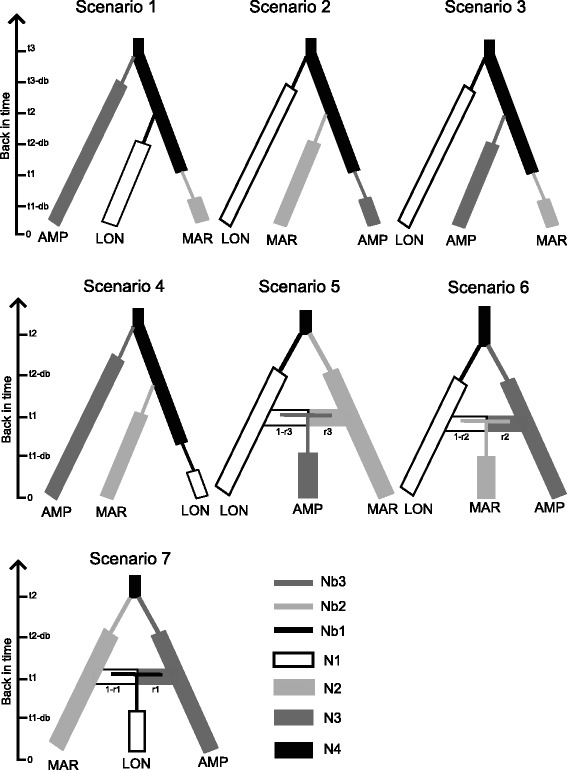
Seven different scenarios inferred for the introduction history of *Xenopsylla cheopis* in Madagascar and Mayotte. Three populations were considered: population from Longoni in Mayotte (LON), population from Marolaka (MAR) and population from Amparafaravola (AMP) in Madagascar where N1, N2, N3 are their respective effective population sizes. Nb1, Nb2 and Nb3 correspond to the numbers of founders in the introduced population. Time scales corresponding to generations back in time from the sampling date (time 0) are shown at the left (t1, t2 and t3 generations ago); *db*, is the duration of the initial bottleneck. For all the scenarios, LON, MAR and AMP derived from an unsampled ancestral population having N4 effective population size. Particularly for each of the scenarios 1–4, derivation from the ancestral population was independent. *Scenario 1*: AMP derived from the ancestral population at t3 followed by LON at t2 then, MAR at t1. *Scenario 2*: LON derived from ancestral population at t3 followed by MAR at t2 then, AMP at t1. *Scenario 3*: LON derived from ancestral population at t3 followed by AMP at t2 then, MAR at t1. *Scenario 4*: AMP derived from ancestral population at t3 followed by MAR at t2 then, LON at t1. The remaining scenarios (5–7) assumed that two parental populations had diverged from an ancestral population at t2 before they would admix and gave the third population at t1. *Scenario 5*: the parental populations were LON and MAR and their admixture at a rate r3 gave AMP. *Scenario 6*: the parental populations were LON and AMP and their admixture at a rate r2 gave MAR. *Scenario 7*: the parental populations were MAR and AMP and their admixture at a rate r1 gave LON

An approximate Bayesian computation (ABC) analysis was conducted to infer the history of population introduction of *X. cheopis* between Mayotte and Madagascar. DIYABC v.2.0 [51] was used to test seven different scenarios of introduction history (Fig. 2). Three populations were chosen to test these scenarios: one from Mayotte (the port of Longoni) and two from Madagascar (Marolaka located in the coastal region of Mahajanga and Amparafaravola located in the Central Highlands; Fig. 1). Amparafaravola was chosen as the site to represent the Central Highlands as this sample was the most representative of the existing genetic variability. For each scenario and each population, the following demographic parameters were estimated: dates of founding of the different populations (as number of generations) (ti), current effective population size (as number of diploid individuals, Ni), number of founders in the introduced populations (Nbi) and the duration of the initial bottleneck (dbi), which may be considered as a latency phase after each introduction event.

For all demographic parameters, prior distribution ranges were implemented according to current knowledge of *X. cheopis*. The generation time of *X. cheopis* varies between 18 days to 20 months according to biotic and abiotic conditions [52]. Laboratory conditions of 25 ± 2 °C temperature and 70–80% relative humidity stimulate an average 12 generations per year. However, we note that natural populations of *X. cheopis* show seasonal dynamics in both the Central Highlands and Mahajanga [53]. Larval and pupal development times and survival can be affected by temperature and humidity [53] and so, generation times may be longer under field conditions. As these conditions are similar to those in our study sites, time (expressed in generations before sampling) was translated into years assuming 12 generations per year. One million simulations were conducted under each scenario (a total of 7 million generations). Posterior probabilities of each scenario were computed by performing direct approach and a polychotomous weighted logistic regression on the 1% of simulated datasets closest to the observed dataset [54, 55] after linear discriminant analysis on summary statistics [51]. Principal components analysis (PCA) was performed using summary statistics of simulated datasets and of observed dataset to check the suitability of the model (scenarios and prior parameters). Confidence in scenario choice was further tested using additional simulations. This included estimation of the probability of a type-II error (the probability of selecting the chosen scenario when it is not correct). After scenario choice, we proceeded to parameter inference estimated from the modes and 95% confidence intervals (CI) of their posterior distributions.

To determine directional relative migration between the three chosen populations, we used the *divMigrate* function from the R-package *diveRsity* v.1.9.89 [56] using Jost’s D as measure of genetic distance [57] and with a bootstrap value of 1000. *DivMigrate* uses the method described in Sundqvist et al. [58].

## Results

### Genetic variability

Nine microsatellite markers produced PCR amplicons for all samples (XC009, XC037, XC021, SF009, XC013, XC023, XC044, XC016 and XC002; cf. Table 2) and only these nine loci were used in genetic analyses. The number of alleles at these 9 loci ranged from 4 to 11 (Table 2) and the mean number of alleles per locus and per populations (Na) ranged from 1.78 to 4.44 (Table 1). The expected heterozygosity (He) ranged from 0.25 to 0.50 while the observed heterozygosity (Ho) varied from 0.07 up to 0.41. All populations except those from Tsiroanomandidy, Toliara and Farafangana, showed a heterozygote deficit (i.e. He > Ho and thus deviation from expected Hardy-Weinberg equilibrium conditions), and with 7 of the 12 sites having significantly positive F_IS_ values (Hardy-Weinberg exact test; *P* < 0.05) (Table 1). Only one of the 36 locus-pair combinations indicated significant linkage disequilibrium (XC002-XC009; *P* < 0.05). Estimated frequencies of null alleles per locus per population ranged from 0.16 to 0.45 (5 cases ≤ 0.20; 9 cases between 0.20 and 0.30; 2 cases between 0.30 and 0.40; and 1 case > 0.40) (Additional file 2: Table S1).

### Population structure

**Fig. 3:**
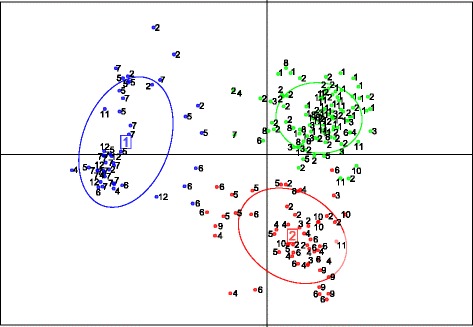
Genetic clusters identified using the DAPC method. Individuals are shown by dots grouped into one of the three genetic clusters. Numbers quoted correspond to the sampling sites: 1, Longoni; 2, Marolaka; 3, Port of Mahajanga; 4, Ankasina; 5, Amparafaravola; 6, Ankazobe; 7, Tsiroanomandidy; 8, Antananarivo; 9, Toliara; 10, Farafangana; 11, Mandritsara; 12, Ambondromisotra

Both Bayesian clustering and multivariate methods identified three main genetic clusters: changes in Ln P(D) approached a plateau at K = 4 (Additional file 3: Figure S1), thus the best K value (the maximum K value observed before the plateau, [43]) was K = 3, the curve of DeltaK [46] showed a slope with a break occurring at K = 3 and the graphical output yielded by DAPC also supported three genetic clusters (Fig. 3).

**Fig. 4:**
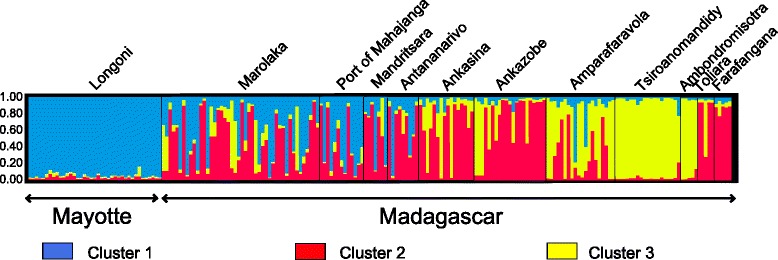
Bar plot showing the genetic structure of individuals from Mayotte and Madagascar generated using STRUCTURE software. Three genetic clusters were assumed (K = 3) and are represented by three colors (*blue*, *red* and *yellow* clusters). Each vertical line represents an individual and the length of each colored line corresponds to the membership coefficient (scale at the left of the bar plot) for a cluster. Individuals are grouped according to their sampling sites

Genetic clusters had some underlying geographical structure, whereby all individuals from Mayotte were assigned to one cluster and individuals from Madagascar were assigned to three clusters including the cluster that contained samples from Mayotte (Fig. 4). All fleas from Mayotte had a membership coefficient > 90% to cluster 1. Fleas from some regions in Madagascar, namely Marolaka, the port of Mahajanga, Mandritsara and Antananarivo, also had substantial membership to cluster 1 as well as a second cluster (indicated in red in Fig. 4) that was only identified in Madagascar. Most individuals from Toliara and Farafangana had high membership coefficients to cluster 2, while fleas from Ankasina and Ankazobe, had membership coefficients that indicated they belonged to cluster 2, but with more apparent genetic influence of the third cluster (indicated in yellow in Fig. 4). Fleas from Tsiroanomandidy and Ambondromisotra presented a membership coefficient greater than 90% to cluster 3; in Amparafaravola, cluster 3 was still dominant though some individuals appeared to belong to cluster 2 or cluster 1 (Fig. 4).

Division of populations into genetic groups was also reflected by the differentiation among these populations suggested by values of F_ST_ (and F_ST_^ENA^; Additional file 4: Tables S2, S3). The F_ST_ values varied from 0 (Tsiroanomandidy *vs* Ambondromisotra) to 0.51 (Antananarivo *vs* Ambondromisotra), with most values quite high (> 0.25) and almost all values of F_ST_ significantly different from 0. The unbiased F_ST_^ENA^ values ranged from 0.01 (Port of Mahajanga *vs* Marolaka) to 0.45 (Antananarivo *vs* Ambondromisotra, Tsiroanomandidy *vs* Longoni and Toliara *vs* Tsiroanomandidy) (Additional file 4: Tables S2, S3). Comparable with estimates of F_ST_, most pairwise estimates of F_ST_^ENA^ were relatively high (> 0.25).

### Population introduction

Using the direct approach, the highest posterior probability (*p*) was obtained for scenario 6 (*p* = 0.50), with scenarios 7 and 5 having substantially lower probabilities (*p* = 0.19 and 0.18 respectively; Additional file 5: Table S4); the logistic approach altered slightly the ranking of demographic scenarios, marginally favoring Scenario 6 (*P* = 0.48) and with Scenario 5 favored over 7 (*P* = 0.43 and 0.06, respectively). All other scenarios for both approaches had low (< 0.1) probabilities (Additional file 5: Table S4 and Additional file 6: Figure S2). The type II error rate (Additional file 7: Table S5) associated with Scenario 6 (probability to select Scenario 6 though it is not correct) was relatively low (*P* = 0.08) providing confidence in the scenario choice. The favored Scenario 6 assumes that the population from Marolaka was a result of an admixture of a population from Longoni and from Amparafaravola (Fig. 2). *DivMigrate* analysis supported Scenario 6 and suggested that asymmetric migrations occurred between flea populations of Mayotte and Madagascar. Flea population sources from Longoni (Mayotte) and Amparafaravola (Madagascar) migrated and constituted the sink population of Marolaka (Madagascar). High significant relative migration rates were obtained: 0.87 (Longoni to Marolaka) and 1 (Amparafaravola to Marolaka) (Additional file 8: Table S6).

Estimates for the different parameters inferred using scenario 6 are given in Table 3. It was estimated that about 47 generations had occurred (95% CI: 16.4–241) between the time of sampling (t0) and the time of population admixture (t1) (Fig. 2; Scenario 6). Assuming 12 generations per year for *X. cheopis* and that the samples were collected in 2014, the population from Marolaka is estimated to have been affected by an introduction that occurred between 1993 and 2012 (t1: 2010 [95% CI: 1993–2012]) at an admixture rate of r2 = 55.8% (95% CI: 21–82%) (Table 3). If fewer generations per year occur under field conditions, a date towards the beginning of this time period is more likely.

**Table 3:** Summary of the DIYABC analysis based on 999,083 simulated datasets averaged over 9990 selected datasets

Parameter names	Prior range^a^	Posterior parameter estimates^a^	95% CI^a^	Relative bias^a^	Relative square root error^a^
Effective population size					
*N*_*1May.*_	10–2000	377	113–1820	0.14	0.36
*N*_2*Mad-Maro.*_	500–15,000	586	654–11,300	-0.65	0.74
*N*_3*Mad-Ampa*_	200–4000	644	394–3720	-0.40	0.47
*N*_4*TGhost*_	100–40,000	3080	2230–23,800	0.44	0.59
*N*_*b1*_	10–2000	673	276–1770	0.11	0.24
*N*_*b2*_	10–2000	1660	674–1930	0.13	0.27
*N*_*b3*_	10–2000	1030	373–1830	0.14	0.27
Time in generation					
*t*_*1*_	2–1000	46.6	16.4–241	0.17	0.31
*t*_*2*_	2–1500	190.0	94.8–1220	0.20	0.31
*d*_*b*_	10–2000	360	113–1860	0.57	0.76
Genetic parameters (rate)					
μ_mic_	1.00 × 10^-5^–1.00 × 10^-3^	1.00 × 10^-4^	1.00 × 10^-4^–2 × 10^-4^	-0.09	0.30
*p*_*mic*_	1.00 × 10^-1^–9.00 × 10^-1^	9.00 × 10^-1^	5.05 × 10^-1^–9.00 × 10^-1^	0.13	0.53
*sn*_*mic*_	1.00 × 10^-8^–1.00 × 10^-5^	1.00 × 10^-8^	1.15 × 10^-8^–3.39 × 10^-6^	-0.99	0.99
*r2*	0.001–0.999	0.558	0.210–0.817	-0.022	0.37

^a^Calculated for the best scenario (scenario 6)

## Discussion

Plague is a health problem in Madagascar and there is a need to understand the natural organization and dynamics of the key vector, *Xenopsylla cheopis*. This first population genetic analysis of *X. cheopis* from Madagascar and Mayotte makes important advances in our understanding: *Xenopsylla cheopis* populations are genetically and geographically structured in Madagascar, with interesting differences compared to previous studies of the principal host *R. rattus* and, a recent population migration of this flea occurred from Mayotte to Madagascar.

### Spatial genetic structure of fleas in Madagascar

Within Madagascar, although there was fairly high genetic differentiation between populations, there was evidence of some gene flow between populations. As predicted, *X. cheopis* appears to show stronger population genetic structure than rats, with three clusters present in Madagascar compared to two for rats [23], and higher F_ST_ values (most values >0.25), compared with a range of 0.01–0.21 for rats [23]. Although these patterns may suggest more limited dispersal in the parasite compared to the host, other factors such as lower effective population size may also play a role [31, 32]. Moreover, as several populations showed shared membership of different genetic clusters and the best-supported scenarios from the ABC analysis (Scenarios 6, 7 and 5) assume population admixture, whilst flea populations are genetically structured, there is also clear evidence of gene flow among populations.

Thus, comparisons of our results for *X. cheopis* with the results for *R. rattus* reveal some similarities and some interesting differences. It is notable that the *R. rattus* genetic structure appears to broadly follow a north/south divide [23]. The northern genetic type of rats was largely restricted to the far north, with STRUCTURE analysis revealing only three sites out of 35 with evidence of mixed ancestry for some individuals. These three sites were geographically located between the northerly and southerly sites and included Mahajanga and a site close to Mandritsara, both sites in our study of *X. cheopis* which showed shared membership of different clusters. Although our results therefore suggest some congruence with the phylogeographic structure of hosts, as suggested for another fur flea, *Listropsylla agrippinae*, in South Africa [33], in general gene flow in *X. cheopis* did not appear to be strongly associated with geographical distance and *R. rattus* genetic structure. Specifically, some sites further south also showed shared membership of different clusters (Antananarivo, Amparafaravola), whilst, unlike for *R. rattus* [23], different flea populations within the Central Highlands could differ substantially in their proportion of membership to different clusters (e.g. Ankazobe *vs* Tsiroanomandidy and Ambondromisotra), indicating that in some cases little gene flow occurs between populations separated by relatively short distances. These apparently contradictory results could be explained by differences in the relative importance of human-mediated dispersal between *R. rattus* and *X. cheopis* and between sites.

A number of factors may lead to more frequent successful dispersal of fleas than black rats. Individual rats may carry a large number of fleas (in a study of rural highland villages in Madagascar, the average number of fleas on rats in houses was >2 [18]). Flea eggs, larvae and pupae are found in dust and debris from activities such as rice pounding [5], suggesting stages other than adults may also be potentially dispersed by human activity. Moreover, the competitive advantage of residents relative to migrants may be more significant for rats than fleas. Critically though, in urban sites that are linked by major roads and a high frequency of human movements, and are therefore more likely to experience human-mediated dispersal, *R. rattus* have largely been replaced by *R. norvegicus* (e.g. Mahajanga and Antananarivo [22]). Thus, at least in the last 30–40 years [22], human-mediated dispersal of *X. cheopis* may be much higher than for *R. rattus*. In contrast, dispersal of *R. rattus* and fleas in more remote, rural sites may be dominated by non-human mediated short-distance dispersal events. As *R. rattus* is abundant and widespread, occurring in diverse habitats [18], this would favor cumulative high levels of gene flow across rural landscapes. Indeed, in a population genetic study of rats at a more local landscape scale, rat populations showed only weak genetic differentiation between adjacent villages, with genetic structure at least partly related to topographic relief [59]. In contrast, the restriction of *X. cheopis* to rats and other peridomestic mammals living inside houses is likely to mean that successful, non-human mediated dispersal between villages is relatively rare.

Whilst fleas did show more genetic structuring than rats, given the patchy distribution of *X. cheopis* in rural landscapes and the likely effects on dispersal rates and effective population sizes, the number of clusters detected in Madagascar (three) is perhaps surprisingly low. This is especially true when compared to the more significant, local clustering of *Y. pestis* [24], despite the more widespread distribution of *Y. pestis* in the landscape (transmitted between rats in habitats other than houses by the endemic flea *S. fonquerniei*) and presumably increased opportunities for dispersal. Although our results do suggest significant levels of gene flow, likely via human-mediated dispersal, they may also reflect the restricted nature of our study as sampling too few individuals per site can lead to underestimation of the number of clusters [60] and incorrect cluster assignment [61]. However, simulation studies have indicated that reasonably limited sampling (6–10 individuals per site) can detect cryptic population structure and that any effects of low sample size are less with hierarchical population structure [60]. In our study, some sites in the south had low sample size from few hosts (Ambondromisotra, Toliara and Farafangana). Despite this, whilst Ambondromisotra had high membership coefficient to cluster 3 and Farafangana to cluster 2, Toliara showed a mix of individuals with high membership coefficient to clusters 2 and 3.

Other factors may also influence the observed patterns of genetic variation within and between populations. Nine among twelve populations of *X. cheopis* showed heterozygote deficit which is significant in almost all cases. Evolutionary processes such as genetic drift, natural selection, inbreeding, mutation, population bottleneck or gene flow may influence allele frequencies and cause heterozygote deficit. Several of these may have contributed to our results. In addition to the gene admixture and gene flow between populations discussed above, fleas obtained from individual rats or rat families may be related, whilst population bottlenecks could occur within the spatially restricted populations of *X. cheopis* or due to seasonal dynamics in highland populations [53]. Increasing the number of sites at both the national scale and landscape scale, and standardizing the sample size within sites, would undoubtedly clarify the population structure of *X. cheopis* within Madagascar and elucidate the role of rat dispersal and human-mediated dispersal. However, our rather limited study already yields important insights, possibly due to the strong hierarchical structure inherent within parasite populations.

### Flea exchange between Mayotte and Madagascar

Our results indicate that limited flea exchange does occur between Mayotte and north Madagascar, with both the ABC analysis and the *divMigrate* analyses suggesting asymmetric gene flow from Mayotte to Madagascar. This exchange is likely to be linked to commerce and shipping routes in the Indian Ocean and reflects similar patterns in previous studies of other species. The mitochondrial haplotype group associated with the *R. rattus* introduction to north Madagascar [23] has previously been found in East Africa and Grande Comore, whilst the mitochondrial haplotypes associated with the southern *R. rattus* introduction was found in Mayotte [62]. This led to the conclusion that Mayotte was colonized by *R. rattus* from Madagascar [62]. Interestingly, although we find evidence of a relatively recent successful introduction of *X. cheopis* from Mayotte, only one *R. rattus* individual in Madagascar has been shown to carry a mitochondrial haplotype related to European human colonization [62], again possibly reflecting a greater success of flea migrants compared to rat migrants as discussed above. Successful introduction is conditioned by the introduction of some rats carrying fleas that are able to successfully reproduce.

### Relevance of findings for plague introduction and dynamics

Our findings are important for assessing the epidemiological risk of plague introduction in plague free areas such as Mayotte. Mayotte already supports populations of both a plague-competent vector species (*X. cheopis*) and plague-competent mammal host species such as *R. rattus* and an insectivore found to be infected with *Y. pestis* in Mahajanga (*Suncus murinus*) [22]. Although port cities such as Mahajanga or Longoni are hubs allowing plague introduction and intercontinental spread [63], the infrequent movement of fleas (and presumably their hosts) between the two ports indicated by this study suggest that the risk is real but limited. As there have been no plague outbreaks in humans in Mahajanga after the outbreaks in 1990s [14] and genetic studies of *Y. pestis* indicate that Mahajanga outbreaks were triggered by dispersal of infected rodents or fleas from the Central Highlands [63], the risk may have been further reduced because plague is no longer circulating in this region. However, studies of the peridomestic mammal and flea communities in Mahajanga are needed to assess this.

Even if plague did arrive in Mayotte due to the movement of infected fleas or hosts, many other factors would influence plague establishment and the risk of epidemics in the human population. One such factor is vector competence. Although the genetic differentiation among populations of *X. cheopis* is not associated with morphological differences, genetic differences might affect vector competence or resistance to pathogens [64] and therefore impact on disease transmission. Correlation between genetic structure of the insect vector and heterogeneity of vector competence has been reported for other disease-vector systems, for example *Aedes albopictus* (insect vector of dengue and yellow fever viruses) [65–67]. Interestingly, Tsiroanomandidy and Ambondromisotra, where cluster 3 occurs, are ‘plague focus’ areas where outbreaks occur every year. More work would need to be conducted to determine if different natural populations of *X. cheopis* differ in their vector competence.

Clearly, to further understand the risk of plague introduction and establishment in neighboring countries such as Mayotte as well as plague outbreaks in Madagascar outside the plague focus, there is a need for further, more extensive studies of flea and rat dynamics and dispersal, including for the endemic vector species, *S. fonquerniei*.

## Conclusion

This study shows strong spatial structure among populations of the flea vector of plague, *X. cheopis*, from Madagascar and the nearby island of Mayotte. Gene flow occurred between Madagascar and Mayotte, but with evidence for a flea population having been introduced recently from Mayotte to Madagascar. As *X. cheopis* is the main vector of plague in Madagascar, the introduction of individuals to Mayotte may present a risk of plague introduction to this island.

## Additional files

Additional file 1: Protocol describing microsatellite primers development (partial genomic libraries enrichment). (DOCX 19 kb)

Additional file 2: Table S1. Estimated frequencies of null alleles per locus per population using Van Oosterhout’s method. (XLSX 10 kb)

Additional file 3: Figure S1. Identification of the number of genetic clusters using the methods of Pritchard & Evanno [43, 46]. (**a**) Posterior probability L(K) and (**b**) DeltaK obtained based on K numbers of genetic populations ranging from 1 to 10. (PDF 52 kb)

Additional file 4: Table S2. Pairwise *F*_ST_ and values between all populations. **Table S3.** Pairwise *F*_ST_^ENA^ values among populations. (XLSX 12 kb)

Additional file 5: Table S4. Probability for each of the seven tested scenarios of population introduction using direct and logistic approaches. (XLSX 9 kb)

Additional file 6: Figure S2. Raw results obtained for the seven scenarios tested using DIYABC software. (**a**) Posterior probabilities of scenarios obtained through a logistic regression computed every 10% (between 10 and 100%) of the number of selected datasets. (**b**) PCA plot allowing to visualize how close datasets simulated (each small dot) under each scenario (different colors) are from the observed dataset (large yellow dot). The most relevant scenario chosen was scenario 6. The probabilities for each scenario using direct and logistic approaches are given. (PDF 786 kb)

Additional file 7: Table S5. Confidence in scenario choice (using posterior based error computations). Pseudo-observed datasets (pods) were drawn from 500 simulated datasets closest to the observed dataset (s = 500). (DOCX 17 kb)

Additional file 8: Table S6. Bidirectional estimate of relative migration rates. The data give relative migration values by pairwise comparisons of the three populations from Madagascar and Mayotte. Analysis was performed using the function *divMigrate* from the package diveRsity [56]. Jost’s D statistic was used to measure the genetic distance [57] and the bootstrap replicates were set to 1000. (XLSX 9 kb)

## Abbreviations

ABC: Approximate Bayesian computation
DAPC: Discriminant analysis of principal components
ENA: Excluding Null Allele
MCMC: Markov Monte Carlo Chain
PCA: Principal components analysis
