# Supplementary material for: Genetic structure and gene flow of the flea Xenopsylla cheopis in Madagascar and Mayotte
Source: Parasit Vectors. 2017 Jul 20;10:347. doi: 10.1186/s13071-017-2290-6 (PMC5520349; doi:10.1186/s13071-017-2290-6)
Supplement: Supplementary file 1 — Protocol describing microsatellite primers development (partial genomic libraries enrichment). (DOCX 19 kb) [file 13071_2017_2290_MOESM1_ESM.docx]

**Appendix S1: Protocol for the partial genomic libraries enrichment**

Briefly, about 9ug DNA was digested with 40 U Sau3AI (Boehringer-Mannheim) and then ligated to 50 pM phosphorylated linkers (SauLA 5’-GGC CAG AGA CCC CAA GCT TCG-3’ annealed to SauLB 5’-PO_4_-GAT CCG AAG CTT GGG GTC TCT GGC C-3’ [1] using 40 U T4 DNA ligase (Promega). The 400-1200bp size fraction was then excised from a 1.8% agarose gel and purified using QIAquick gel extraction kit (Qiagen). The DNA fragments were hybridized with M2-80 streptavadin-coated magnetic beads (Dynal) that had been incubated with 3’-end biotin-labelled (CA)_12_ and (CAA)_8_ oligonucleotides (MWG, Biotech). After a series of differential stringency washes in 2xSSC and 1xSSC, 8ul of the enriched DNA was made double stranded and amplified in a 50 μl PCR (2X ReddyMix (Abgene), 30 pmol primer SauLA). The thermal profile of the PCR was: 95**°**C 5 min, 30x[95**°**C 50 s, 56**°**C 1 min, 72**°**C 2 min], 72**°**C 10 min. PCR products were purified using a QIAquick PCR purification kit (Qiagen), ligated into pGEM-T vector (Promega) and transformed into JM109 *E. coli* competent cells (Promega). Recombinant clones were identified using black/white screening on S-gal (Sigma) agar/ampicillin plates. Plasmids containing an insert with a microsatellite were identified by two or more amplified products after PCR primed with 37.5 pmol SauLA and 18.8 pmol of (nonbiotinylated) microsatellite oligonucleotide (CA)_12_ (see [2]). Putative positive clones were cycle sequenced using Big Dye^TM^ chemistry (Applied Biosystems) and electrophoresis on an ABI3130xl (Applied Biosystems). Primers flanking microsatellite regions were designed using Primer3 v.0.4.0 [3] ([http://frodo.wi.mit.edu](http://frodo.wi.mit.edu/)).

We sequenced 243 putative positive clones, of which 216 (89%) contained repetitive motifs greater than 6 repeat units. However, primers could only be developed around 44 of these loci. Seventeen were dropped because of consistent PCR-failures or multiple/spurious bands produced during first PCR screenings, leaving 27 loci for genotype testing.

These 27 loci were tested for polymorphisms using samples of fleas from the Central Highlands of Madagascar (n~8) and a tailed primer method to label the PCR products [4]. In this method forward primers are synthesized with a 5’ sequence of a primer that is labeled with either 6-FAM, NED, PET or VIC fluorophores (Applied Biosystems). The 10ul PCR consisted of 5μl 2X ReddyMix (Abgene), 2mM MgCl_2_, 10ug BSA, 5-50ng template DNA, 4pmol of each of the tailed, reverse and labeling primer. PCR products were pooled with Genescan 500 liz (Applied Biosystems), separated by electrophoresis on an ABI3130*xl* and sized using GeneMapper software (Applied Biosystems). At this stage 11 loci were selected as the most promising based on apparent levels of polymorphism and ease of scoring (*e.g*. no, few stutter bands). In addition, one microsatellites locus isolated (using the same enrichment method) from the endemic Madagascar flea *Synopsyllus fonquerniei* (S. Telfer, unpublished data), that consistently worked in *X. cheopis* and was apparently polymorphic, was included in the final panel of 12 microsatellite loci used for genotyping in this study (Table 1).

References:

1. Refseth UH, Fangan BM, Jakobsen KS. Hybridization capture of microsatellites directly from genomic DNA. Electrophoresis. 1997;18(9):1519-23.

2. Gardner MG, Cooper SJB, Bull CM, Grant WN. Isolation of microsatellite loci from a social lizard, *Egernia stokesii*, using a modified enrichment procedure. J Hered. 1999;90(2):301-04.

3. Rozen S, Skaletsky H. Primer3 on the WWW for general users and for biologist programmers. In: Misener S, Krawetz SA, editors. Bioinformatics Methods and Protocols: Humana Press; 1999. p. 365-86.

4. Schuelke M. An economic method for the fluorescent labeling of PCR fragments. Nat Biotechnol. 2000;18(2):233-34.
