# Supplementary figures and images for: Genetic structure and gene flow of the flea Xenopsylla cheopis in Madagascar and Mayotte
Source: Parasit Vectors. 2017 Jul 20;10:347. doi: 10.1186/s13071-017-2290-6 (PMC5520349; doi:10.1186/s13071-017-2290-6)

(a)

$L(K)$  (mean  $\pm$  SD)

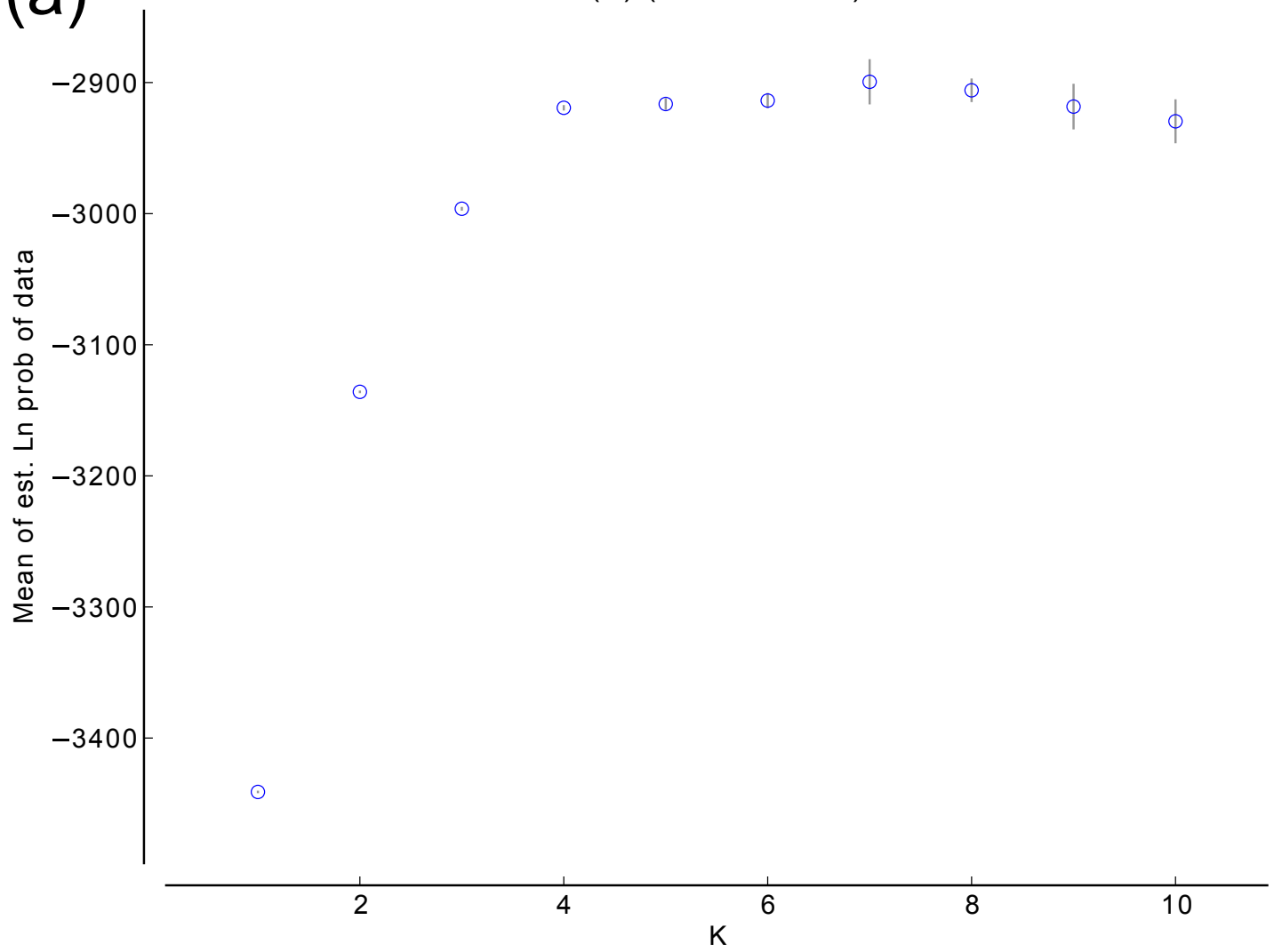

(b)

$\Delta K = \text{mean}(|L''(K)|) / \text{sd}(L(K))$

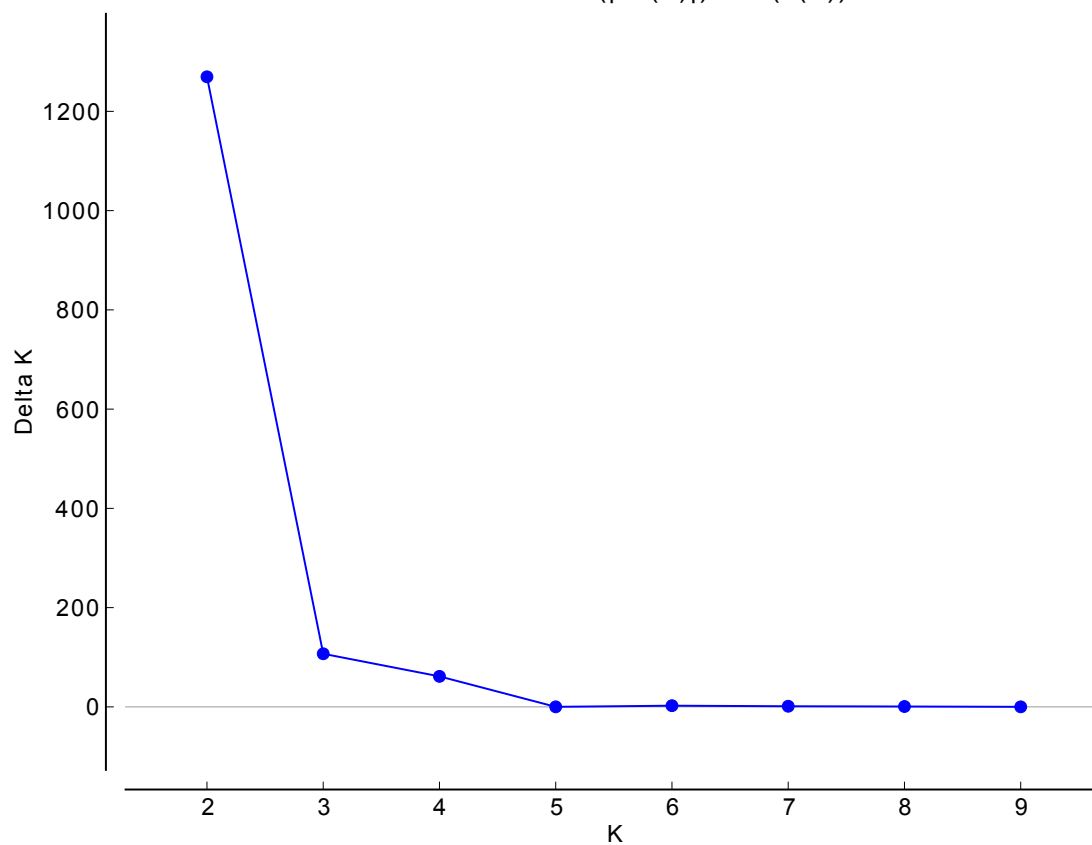

Supplement: Supplementary file 3 — Identification of the number of genetic clusters using the methods of Pritchard & Evanno [43, 46]. (a) Posterior probability L(K) and (b) DeltaK obtained based on K numbers of genetic populations ranging from 1 to 10. (PDF 52 kb) [file 13071_2017_2290_MOESM3_ESM.pdf]

(a)

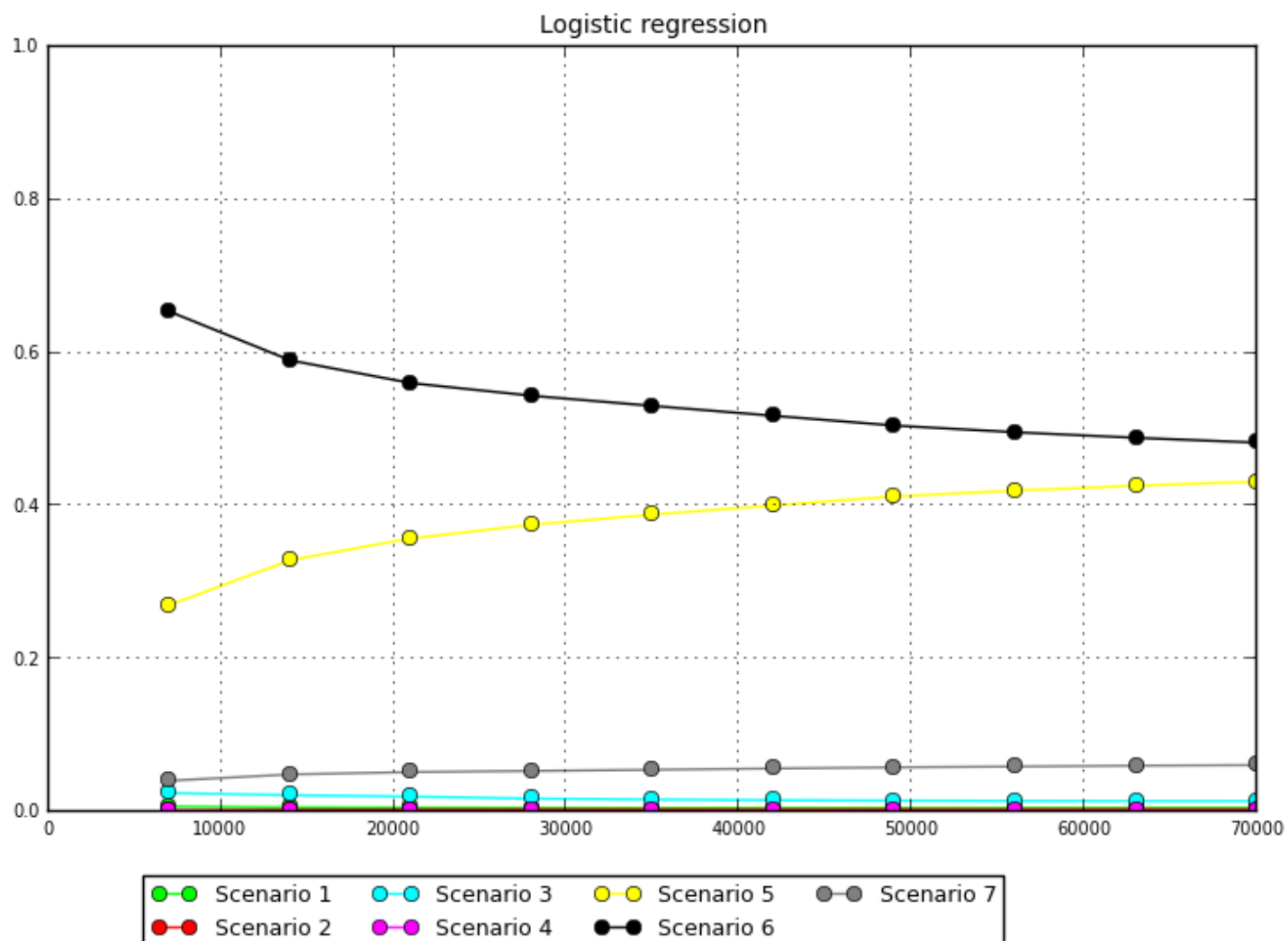

(b)

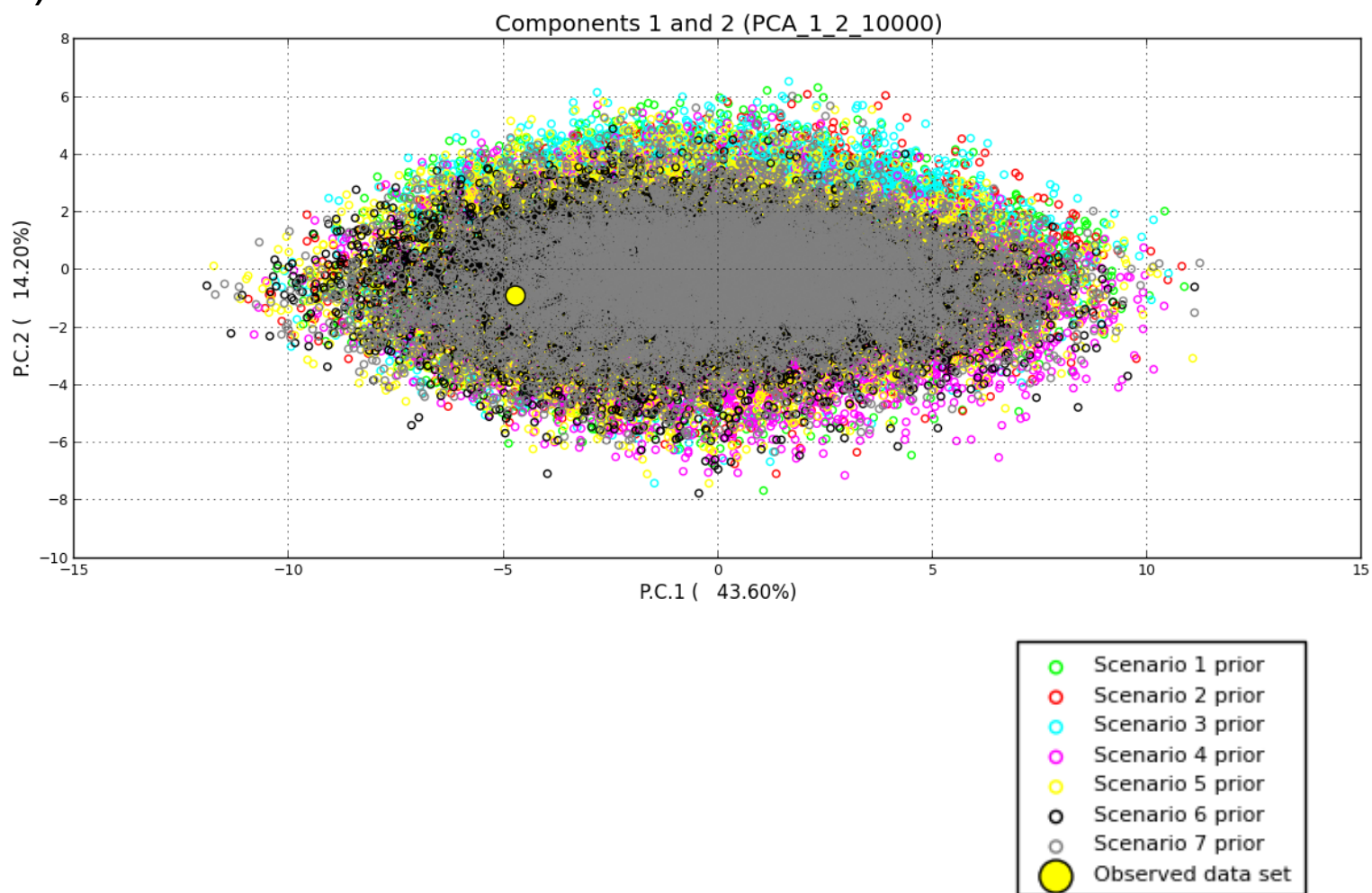

Supplement: Supplementary file 6 — Raw results obtained for the seven scenarios tested using DIYABC software. (a) Posterior probabilities of scenarios obtained through a logistic regression computed every 10% (between 10 and 100%) of the number of selected datasets. (b) PCA plot allowing to visualize how close datasets simulated (each small dot) under each scenario (different colors) are from the observed dataset (large yellow dot). The most relevant scenario chosen was scenario 6. The probabilities for each scenario using direct and logistic approaches are given. (PDF 786 kb) [file 13071_2017_2290_MOESM6_ESM.pdf]
