# Supplementary material for: Genetic structure and gene flow of the flea Xenopsylla cheopis in Madagascar and Mayotte
Source: Parasit Vectors. 2017 Jul 20;10:347. doi: 10.1186/s13071-017-2290-6 (PMC5520349; doi:10.1186/s13071-017-2290-6)
Supplement: Supplementary file 7 — Confidence in scenario choice (using posterior based error computations). Pseudo-observed datasets (pods) were drawn from 500 simulated datasets closest to the observed dataset (s = 500). (DOCX 17 kb) [file 13071_2017_2290_MOESM7_ESM.docx]

**Additional file 7: Table S5.** Confidence in scenario choice.

|  |  | Scenario with highest posterior probability | | | | | |  | Probability to detect  scenario 6 while data  were generated  using scenario i |
| --- | --- | --- | --- | --- | --- | --- | --- | --- | --- |
|  |  | 1 | 2 | 3 | 4 | 5 | **6** | 7 |  |
| Simulated under scenario | 1 | 354 | 10 | 15 | 69 | 2 | **29** | 21 | 0.058 |
|  | 2 | 20 | 349 | 96 | 5 | 10 | **15** | 5 | 0.03 |
|  | 3 | 69 | 69 | 329 | 0 | 10 | **23** | 0 | 0.046 |
|  | 4 | 171 | 5 | 0 | 239 | 0 | **58** | 27 | 0.116 |
|  | 5 | 17 | 14 | 27 | 21 | 294 | **81** | 46 | 0.162 |
|  | 6 | 43 | 8 | 17 | 37 | 25 | **335** | 35 |  |
|  | 7 | 22 | 3 | 1 | 83 | 26 | **77** | 288 | 0.154 |

Total type 2 error of scenario 6 = 0.08

Total type 1 error of scenario 6 = 0.33

The posterior probabilities of each pseudo-observed dataset produced were estimated using simulated datasets and the proportion of times the right scenario (scenario 6) has the highest posterior probability was measured (integer numbers in the table). The right column of the table gives the probability to detect scenario 6 while data were generated using the all other scenarios. Total type 1 and 2 errors of scenario 6 are given below the table.
